# The Potential for Reassortment between Oropouche and Schmallenberg Orthobunyaviruses

**DOI:** 10.3390/v9080220

**Published:** 2017-08-11

**Authors:** Natasha L. Tilston-Lunel, Xiaohong Shi, Richard M. Elliott, Gustavo Olszanski Acrani

**Affiliations:** 1 MRC-University of Glasgow Centre for Virus Research, 464 Bearsden Road, Glasgow G61 1QH, UK; Xiaohong.Shi@glasgow.ac.uk (X.S.); natasha.tilston@gmail.com (R.M.E.); 2 Biomedical Sciences Research Complex, School of Biology, University of St Andrews, St Andrews KY16 9ST, UK; 3 Department of Microbiology, National Emerging Infectious Disease Laboratories, Boston University School of Medicine, Boston, MA 02118, USA; 4 Universidade Federal da Fronteira Sul, Campus Passo Fundo. Rodovia RS 153 - Km 03, Jardim América, CEP 99034-600 Passo Fundo—RS, Brasil

**Keywords:** Oropouche virus, Schmallenberg virus, bunyavirus, *Orthobunyavirus*, Simbu serogroup, minigenome, reassortment

## Abstract

A number of viruses within the *Peribunyaviridae* family are naturally occurring reassortants, a common phenomenon for segmented viruses. Using a minigenome-reporter and virus-like particle (VLP) production assay, we have accessed the potential of Oropouche virus (OROV), Schmallenberg virus (SBV), and other orthobunyaviruses within the Simbu serogroup to reassort. We found that the untranslated region (UTR) in the medium segment is a potential contributing factor for reassortment by the tested viruses. We demonstrate that for promoter activity to occur it was essential that the viral RNA polymerase (L) and nucleocapsid (N) proteins were from the same virus, reinforcing the hypothesis that the large and small segments that encode these proteins segregate together during genome reassortment. Our results indicate that, given the right epidemiological setting, reassortment between SBV and OROV would potentially be feasible and could contribute to the emergence of a new Simbu virus.

## 1. Introduction

The *Orthobunyavirus* genus in the *Peribunyaviridae* family (order *Bunyavirales*) comprises at least 30 viruses that can cause disease in humans and animals, including Oropouche virus (OROV), Schmallenberg virus (SBV) and La Crosse virus (LACV) [1,2]. The *Orthobunyavirus* genome is tripartite, made up of single-stranded segments of negative-sense polarity. The small genome segment encodes the nucleocapsid (N) protein and, in a majority of viruses, an additional nonstructural protein called NSs from an overlapping reading frame. The medium segment encodes a protein precursor for two envelope glycoproteins (Gn and Gc) and a nonstructural protein called NSm. The large genome segment encodes the RNA-dependent RNA polymerase (L protein). The 5′ and 3′ ends of these genome segments are flanked by highly conserved and complementary genus-specific sequences, which contain signals for genome transcription, replication, and packaging. Each genomic segment in combination with the N and L proteins form a functional ribonucleoprotein (RNP) [2,3,4,5,6].

The segmented nature of the bunyavirus genome potentially allows for easy reassortment between its different members, a fact that was eloquently proposed a few years ago [7]. This phenomenon of viral reassortment is well documented for viruses with segmented-genomes and occurs when two genetically related viruses co-infect the same host cell. Known orthobunyavirus reassortants include Apeu virus [8], Aino virus [9], Ngari virus [10], Iquitos virus [11], Madre de Dios virus [12] and Perdoes virus [13]. Bunyavirus reassortants tend to contain small and large segments from the same parental virus, whilst the medium segment donor remains, in most cases, unknown. Since the small and large segments encode the N protein and the viral polymerase (L), which are essential for genome replication, it is likely that they also co-evolve together [4,14]. The medium segment, on the other hand, encodes the Gn and Gc, which bind to the attachment and entry receptors on the host cell. Progeny viruses that arise from medium segment reassortment would therefore contain new genetic information, potentially changing the tissue and/or host tropism of that virus [4,7].

To investigate whether OROV and SBV, both important pathogens in the *Orthobunyavirus* genus, could reassort, we utilised a minigenome and virus-like particle assay to test their protein-protein and protein-RNA interactions. OROV and SBV are both midge-borne and are classified in the Simbu serogroup. Whilst OROV causes a febrile illness in humans in South America [15,16], SBV is responsible for ruminant fetal malformations in Europe [17,18]. Separated by host and geographic distance, these viruses are ideal for studying the determinants of cross-species transmission. Our study also demonstrates how a combination of minigenome and virus-like particle assays can be used to predict reassortment amongst bunyaviruses, without the requirement to generate infectious virus.

## 2. Materials and Methods

### 2.1. Cells

BSR-T7/5 cells, which stably express T7 RNA polymerase [19] were grown in Glasgow minimal essential medium (GMEM, Invitrogen, UK) supplemented with 10% fetal bovine serum (FBS, Invitrogen), 10% tryptose phosphate broth (TPB, Invitrogen), and 1 mg/mL G418 (Geneticin, Invitrogen). BHK-21 cells were grown in GMEM supplemented with 10% newborn calf serum (NCS, Invitrogen) and 10% TPB. Both cell lines were grown and maintained at 37 °C with 5% CO_2_.

### 2.2. Plasmids

Minigenome-expressing and protein-expressing plasmids for OROV, SBV, and Bunyamwera virus (BUNV) have been previously described [6,18,20,21,22]. Plasmids pTM1OROV-L, pTM1OROV-M, and pTM1OROV-N for OROV; pTM1SBV-L, pTM1SBV-M and pTM1SBVN for SBV; and pTM1BUNV-L, pTM1BUNV-M and pTM1BUNV-N for BUNV express the viral polymerase, glycoproteins Gn and Gc, and the N protein under the control of a T7 promoter and the internal ribosomal entry site (IRES), from encephalomyocarditis virus (EMCV) [23]. Nucleotide substitutions to generate OROV M-minigenome-(8A/A 9A/A) and SBV M-minigenome-(8T/A 9C/A) were introduced by QuikChange site-directed mutagenesis (Stratagene, UK). The minigenome plasmids TVT7RSBVLRen(-) and pTVT7AKVMRen(-) were generated as previously described in [20]. The coding sequences of SBV large [18] and Akabane virus (AKV) medium segments (Plasmid donated by Massimo Palmarini, Centre for Virus Research, University of Glasgow, UK) in the pTVT7 plasmids were deleted by excision PCR (KOD Hot Start DNA polymerase; Merck, UK), leaving the untranslated region (UTR) intact and used as a vector to clone in the *Renilla* luciferase coding sequence as in [20]. Plasmids pTVT7OYAMRen(-) and pTVT7PEDMRen(-), which contain Oya virus (OYAV) and Perdoes virus M UTRs, were generated using pTVT7Ren(-) as a template. This plasmid generates T7 transcripts containing *Renilla* luciferase in the negative sense. Oligonucleotides containing the sequences corresponding to either the 5′ UTR or 3′ UTR of OYAV or Perdoes virus were then cloned in with excision PCR (KOD Hot Start DNA polymerase; Merck) and In-Fusion HD Cloning (Clonetech, Takara Bio, UK) techniques. This approach of synthetically cloning in the UTR sequences for OYAV and Perdoes virus was adopted due to the unavailability of viral stocks or plasmids for either virus. Oligonucleotides were based on sequences in GenBank.

### 2.3. Minigenome Assay

5 × 10^4^ BSR-T7/5 cells/mL in 24-well trays were transfected with the desired amount of protein-expressing plasmids, 125 ng of a minigenome-expressing plasmid and 25 ng pTM1-FF-Luc using Lipofectamine 2000 (Invitrogen, UK), as per the manufacturer’s instructions. The amount of DNA in each well was kept constant by the addition of empty vector, pTM1. To normalize transfection efficiencies, cells were co-transfected with firefly luciferase expression plasmid pTM1-FF-luc, allowing induction levels of *Renilla* luciferase to be calculated [21]. At 24 h post-transfection (p.t.), cells were washed with 0.5 mL of phosphate buffered saline (PBS) and then lysed using 100 μL Passive lysis buffer from the Promega Dual-Luciferase Reporter Assay kit (Promega, UK). The rest of the assay was carried out as per the manufacturer’s recommendations. Readings were measured on a GloMax 20/20 luminometer (Promega).

### 2.4. Virus-Like Particle Production Assay

1.5 × 10^5^ BSR-T7/5 cells/mL in 12-well trays were transfected with 500 ng of pTM1-L and pTM1-N, 125 ng of a minigenome-expressing plasmid, and 25 ng pTM1-FF-Luc. At 24 h p.t., *Renilla* and firefly luciferase activities were measured using Dual-Luciferase Reporter Assay kit (Promega). To generate virus-like particles (VLPs), the minigenome transfection mix was supplemented with 200 ng pTM1-M. At 48 h p.t., supernatants were harvested, clarified by centrifugation (1400 *g* for 5 min), digested with benzonase (≥250 units/μL, Novagen, UK), and used to infect either BSR-T7/5 cells that were pre-transfected with 1 μg of pTM1-L, pTM1-N and pTM1-M for 24 h, or untransfected BHK-21 cells. *Renilla* activity was measured after 24 h using the Dual-Luciferase Reporter Assay kit (Promega).

## 3. Results

### 3.1. Schmallenberg virus N and L Proteins Are Capable of Transcribing and Replicating Oropouche Virus Minigenomes

**Figure 1:**
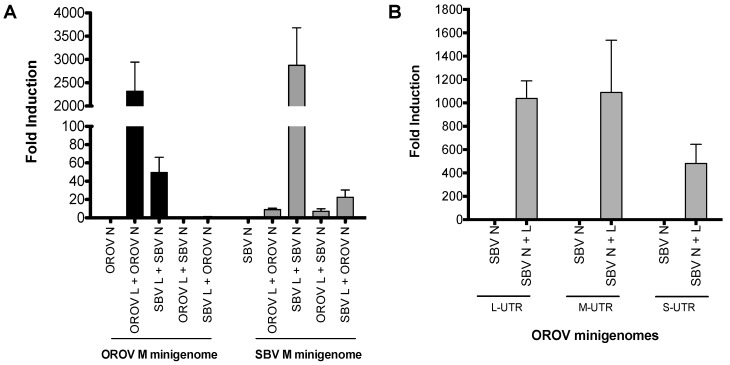
Minigenome activity: (**A**) BSR-T7/5 cells were transfected with pTM1-N and/or pTM1-L (250 ng) and M-minigenome (125 ng) plasmids, pTVT7R-SBVMRen(-), or pTVT7OROVMRen(-). Firefly luciferase-expressing pTM1-FF-Luc (25 ng) served as a transfection control. At 24 h post-transfection (p.t.), *Renilla* and firefly luciferase were measured using a Dual-Luciferase Reporter Assay kit (Promega). Luciferase values were normalized and minigenome-activity expressed as fold-induction over background control (no pTM1-L). Error bars indicate standard deviation (SD; *n* = 3). (**B**) BSR-T7/5 cells were transfected as previously described. 250 ng of pTM1SBV-N and/or pTM1SBV-L were transfected with 125 ng of pTVT7OROVLRen(-), pTVT7OROVMRen(-), or pTVT7OROVSRen(-), and pTM1-FF-Luc (25 ng). At 24 h p.t., *Renilla* and firefly luciferase were measured using a Dual-Luciferase Reporter Assay kit (Promega). Luciferase values were normalized and minigenome-activity expressed as fold-induction over background control (no pTM1-L). Error bars indicate SD (*n* = 3). OROV, Oropouche virus; SBV, Schmallenberg virus; L, viral polymerase; N, nucleocapsid protein; M minigenome, medium segment analogue; L-UTR, large segment untranslated region; M-UTR, medium segment untranslated region; S-UTR, small segment untranslated region.

To assess whether the N and L proteins of SBV recognise and initiate transcription and replication of the OROV minigenome, and vice versa, we compared minigenome activity with a combination of homologous and/or heterologous N and L proteins. To do this, we transfected BSR-T7/5 cells with OROV or SBV medium segment minigenomes (M-minigenome) with either the N and L protein-expressing plasmids of the respective virus or a combination of OROV and SBV N and L (Figure 1A).

The minigenome plasmids contain the *Renilla* luciferase reporter gene flanked by viral UTRs, in the viral genomic (negative) sense, hence functioning as a genome segment analogue. Replication and transcription of this minigenome is initiated only when the N and L proteins can recognise the viral UTR as a functional promoter. This then leads to the expression of *Renilla* luciferase. As expected, minigenome activity was detected in the homologous minigenome systems (i.e., OROV M-minigenome with OROV N-L or SBV M-minigenome with SBV N-L) (Figure 1A). Interestingly, a greater than 50-fold increase in minigenome activity was also detected in OROV M-minigenome with SBV N-L, contrasting a less than 10-fold increase over background in SBV M-minigenome with OROV N-L (Figure 1A). Furthermore, minigenome activity increased when the N and L protein pair came from the same virus, in contrast to when heterologous N and L proteins were used (Figure 1A).

Since SBV N and L could recognise and support OROV M-minigenome transcription and replication, we next tested their ability to utilise OROV small and large segment UTRs via S- and L-minigenomes. As shown in Figure 1B, luciferase activity was detected from all three OROV minigenomes, indicating that SBV N and L proteins were able to recognize the UTRs of all OROV segments (Figure 1B). In short, the minigenome assays suggest that SBV polymerase can interact and recognise OROV UTRs and form a functional heterologous RNP consisting of SBV N and L proteins and OROV minigenome.

### 3.2. Packaging and Assembly of Heterologous Viral-Like Particles

**Figure 2:**
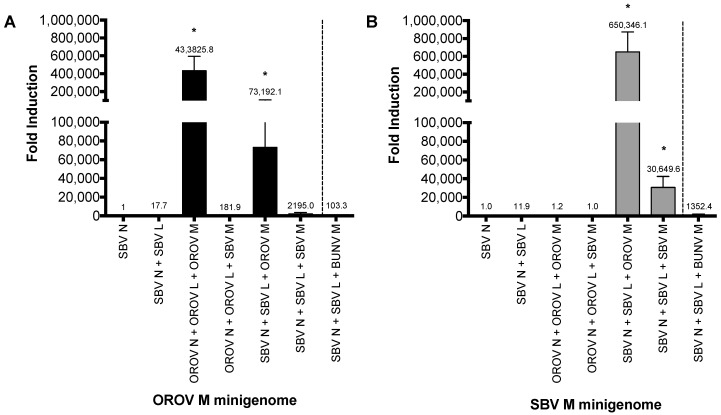
Virus-like particle (VLP) assay. (**A**,**B**) BSR-T7/5 cells were transfected with pTM1SBV-L (200 ng) and pTM1SBV-N (400 ng) or pTM1OROV-L and pTM1OROV-N (400 ng), along with pTM1-M (200 ng), and M-minigenome (200 ng) and pTM1-FF-Luc (40 ng). At 48 h p.t., clarified supernatant was used to infect cells pre-transfected with protein-expressing plasmids only, as indicated. At 24 h p.t., luciferase readings were measured as described above. *Renilla* activity is expressed as fold induction over control pTM1-N. Error bars indicate SD (*n* = 3). * *p* < 0.05 (pair-wise Student’s *t*-test). BUNV, Bunyamwera virus; OROV, Oropouche virus; SBV, Schmallenberg virus; L, viral polymerase; N, nucleocapsid protein; M, glycoprotein precursor; M minigenome, medium segment analogue.

To investigate whether the heterologous RNP can be packaged into viral particles, we examined the formation of infectious VLPs (iVLPs) [22]. Here, BSR-T7/5 cells were transfected with either SBV or OROV M-minigenome in combination with different viral N, L and M cDNA expression plasmids (Figure 2A,B). The family prototype Bunyamwera virus (BUNV) glycoprotein was also included as a control. The formation of iVLP from transfected cells was examined by using that supernatant to infect fresh BSR-T7/5 cells that were pre-transfected with N, L and M plasmids, in order to boost transcription as previously described [24]. Positive samples were further tested on untransfected BHK-21 cells for confirmation (data not shown). As we expected, *Renilla* luciferase was detected from cells infected with OROV and SBV VLP controls (Figure 2A, bar 3 and Figure 2B, bar 6). Consistent with the aforementioned minigenome assay, reporter activity was also detected from cells infected with hybrid VLPs, i.e., OROV glycoproteins-spiked VLPs containing heterologous RNPs (OROV or SBV M-minigenomes with SBV N-L) (Figure 2A,B, bar 5). However, no heterologous iVLP was formed by the SBV glycoproteins with OROV M-minigenome RNP and only low reporter activity was detected from OROV M-minigenome-SBV N-L (Figure 2A,B, bar 4). Interestingly, the VLPs with the OROV glycoprotein spike and SBV RNPs generated a higher signal than SBV’s own VLP system (Figure 2B), which could just be down to an optimization efficiency of the SBV VLP system. A low signal was also observed for BUNV glycoprotein spiked VLP containing OROV or SBV minigenome with SBV N-L (Figure 2).

As the interaction between viral Gn cytoplasmic tail (CT) and N or RNP is crucial for bunyavirus assembly [25], the results indicate that OROV Gn glycoprotein can interact with heterologous SBV RNPs to assemble iVLPs. The efficient formation of infectious heterologous VLPs suggest that the reassortment between OROV and SBV would likely lead to a new reassortant containing a combination of small and large segments from SBV and a medium segment from OROV.

### 3.3. The Importance of Untranslated Region Positions Eight and Nine

**Figure 3:**
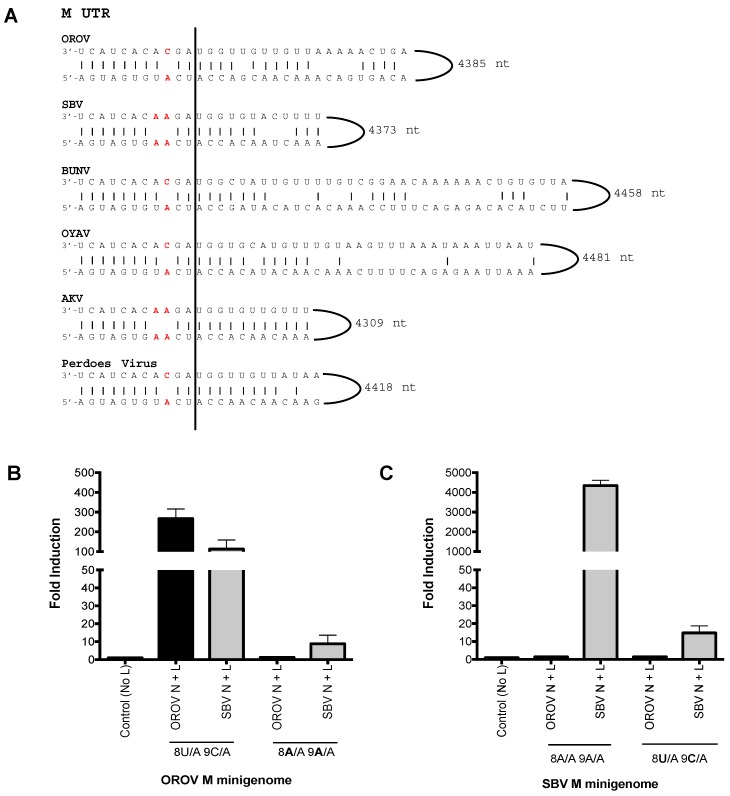
Analysis of the Simbu medium segment untranslated region (M UTR). (**A**) Sequence comparison of the 3′ and 5′ panhandle nucleotides of the M segments of Oropouche virus (OROV) (GenBank accession no, KP052851), Schmallenberg virus (SBV) [18], Bunyamwera virus (BUNV) (GenBank accession no, NC_001926), Oya virus (OYAV) (GenBank accession no, JX983193), Akabane virus (AKV) (GenBank accession no, NC_009895) and Perdoes virus (GenBank accession no, KP691628). OROV, BUNV, OYAV, and Perdoes viruses contain a single mismatch at position nine (8A/U 9C/A), whereas SBV and AKV contain a double mismatch at positions eight and nine (8A/A and 9A/A). These mismatches have been highlighted in red in the sequence alignment. (**B**,**C**) Minigenome assays. Performed as described above. Error bars indicate SD (*n* = 3).

Since SBV N and L proteins were able to recognise OROV M UTR and not the other way around we decided to investigate the sequence difference that may contribute to this UTR recognition. We aligned the medium segment UTRs of OROV and SBV, along with that of other Simbu serogroup viruses—Akabane virus (AKV), Oya virus (OYAV), Perdoes virus, and BUNV (Figure 3A). Interestingly, SBV and AKV (another ruminant pathogen) contain 8A/A-9A/A “double-mismatch” at positions eight and nine of the M UTR. This is a variation from the “typical” 8A/U-9C/A “pairing/mismatch” present in OROV, BUNV, OYAV, and Perdoes virus. These positions have been shown to be important for orthobunyavirus promoter activity [3,20,26]. We next analysed the role these residues may play in promoter recognition by the viral N and L proteins. The eighth and ninth nucleotides of OROV M-minigenome (8A/U–9C/A) were altered to mimic the residues of SBV M-UTR (8A/A–9A/A), resulting in the first 11 residues of OROV M-UTR being changed to those of SBV (Figure 3A).

A minigenome assay with the plasmid bearing this mutation (OROV M-minigenome 8A/A 9A/A) resulted in a loss of UTR functionality with OROV N and L proteins and a decreased efficiency of UTR functionality with SBV N and L proteins (Figure 3B; 8A/A, 9A/A). A similar approach using the SBV M-minigenome was then carried out, where the first 11 residues of SBV M-UTR were altered to mimic OROV M-UTR (8A/U 9C/A), however this change did not result in rescue of luciferase activity for OROV N and L proteins (Figure 3C; 8U/A 9C/A). To test whether the terminal 11 nucleotides alone are sufficient for promoter activity, an OROV M-minigenome containing only the terminal 5′ and 3′ 13 nucleotides was generated, however this minigenome with the truncated UTR was not functional with either OROV or SBV N and L proteins (data not shown), consistent with previous work [27].The length of the 3′ UTR of orthobunyaviruses vary considerably and, amongst the viruses focused on in this study, AKV has the shortest 5′ UTR: only 22 nucleotides. Previous work has demonstrated that the minimum requirement for a viable BUNV S segment mutant is a 22 nucleotide 5′ UTR, with at least 112 nucleotides at the 3′ terminus [28].

### 3.4. OROV N and L Proteins Are Capable of Transcribing and Replicating a Large Segment SBV Minigenome

**Figure 4:**
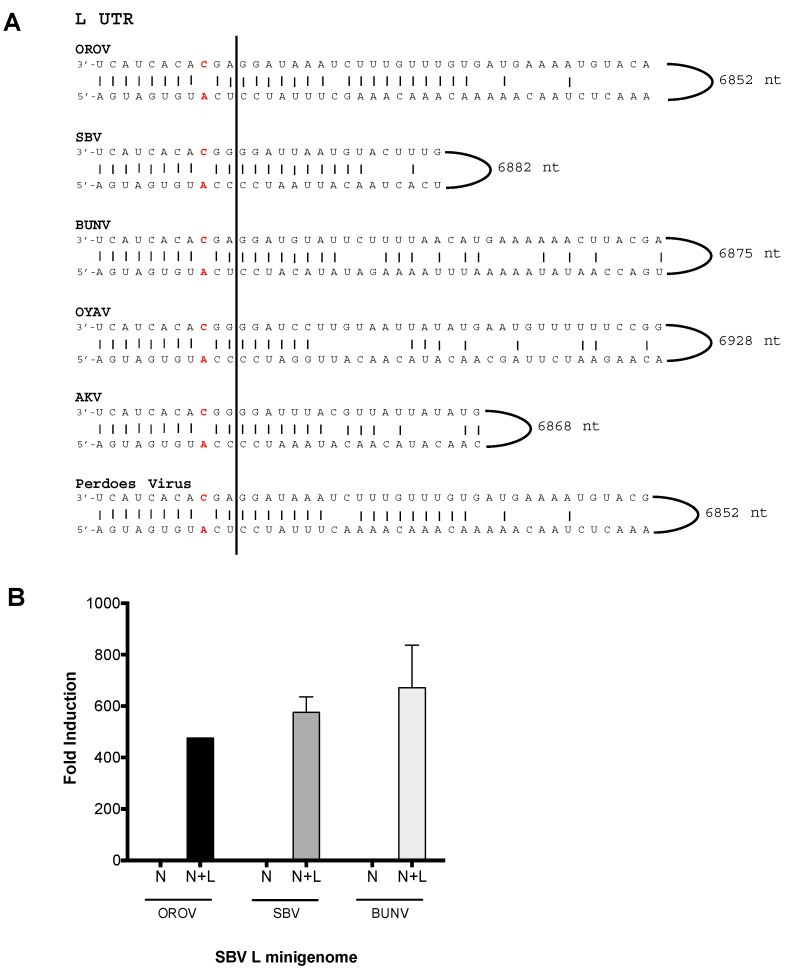
Analysis of Simbu large segment untranslated region (L UTR). (**A**) Sequence comparison of the 3′ and 5′ panhandle nucleotides of the L segments from Oropouche virus (OROV) (GenBank Accession no. KP052850), Schmallenberg virus (SBV) [18], Bunyamwera virus (BUNV) (GenBank Accession no. NC_001925), Oya virus (OYAV) (GenBank Accession no. JX983194), Akabane virus (AKV) (GenBank accession no, NC_009894) and Perdoes virus (GenBank Accession no. KP691627). All viruses contain a single mismatch at position nine (8 A/U 9**C/A**). These mismatches have been highlighted in red in the sequence alignment. (**B**) Minigenome assays. BSR-T7/5 cells were transfected with OROV N-L, or SBV N-L, or BUN N-L (250 ng), and TVT7RSBVLRen(-) (125 ng) and pTM1-FF-Luc (40 ng). At 24 h p.t., luciferase activity was measured and calculated and described above. Error bars indicate SD (*n* = 3).

The large segment UTRs of viruses OROV, SBV, BUNV, AKV, OYAV and Perdoes virus, in contrast to their medium segment UTRs, contain identical nucleotides in the first eleven positions (Figure 4A). The large segment UTR (L-UTR) of SBV and AKV do not contain the 8A/A–9A/A “double-mismatch” at positions eight and nine that are present in their M UTR (Figure 3A). Instead the L UTR of SBV and AKV possess the “typical” 8A/U 9C/A “pairing/mismatch”, similar to OROV, BUNV, OYAV and Perdoes virus (Figure 4A). Thereby, we tested the SBV L segment promoter in a minigenome assay with OROV or BUN N and L proteins. Interestingly, OROV and BUNV N and L were now able to transcribe and replicate this SBV L-minigenome (Figure 4B). These results demonstrate that, in contrast to SBV M-UTR (Figure 1A), the N and L proteins of OROV are capable of recognising and using SBV L-UTR as a promoter.

### 3.5. Analysis of the Reassortment Potential amongst Various Simbu Serogroup Viruses

**Figure 5:**
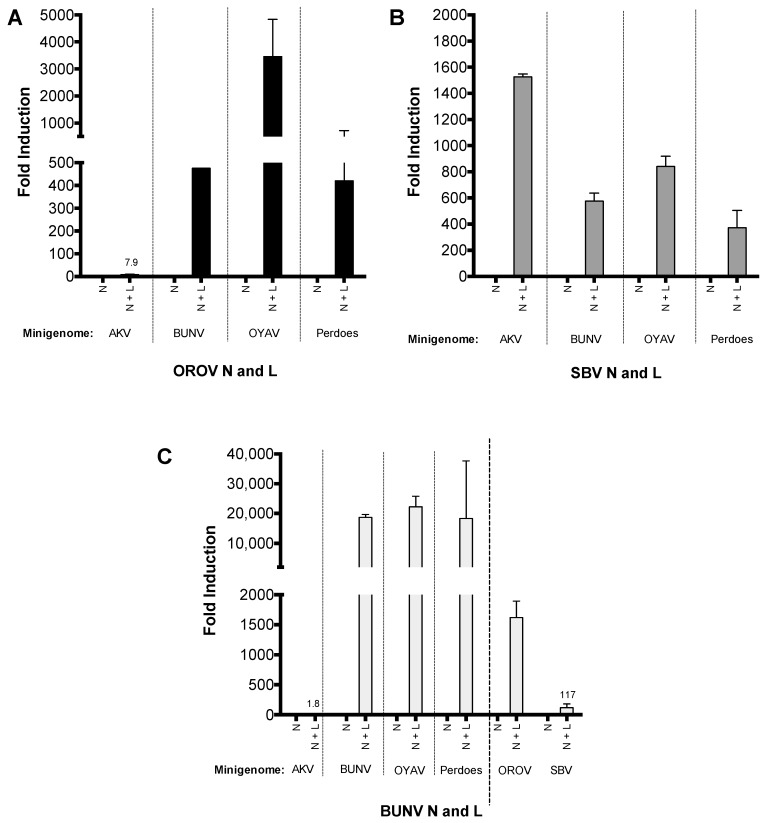
Simbu M-minigenome comparison (**A**–**C**) BSR-T7/5 cells were transfected with pTM1-N and/or pTM1-L (250 ng) and M-minigenome (125 ng) plasmids. Firefly luciferase-expressing pTM1-FF-Luc (25 ng) served as a transfection control. *Renilla* and firefly luciferase were measured using a Dual-Luciferase Reporter Assay kit (Promega) 24 h p.t. Luciferase values were normalized and minigenome-activity expressed as fold-induction over background control (no pTM1-L). Error bars indicate SD (*n* = 3).

To further investigate the importance of the medium segments UTR in genomic reassortment, we generated medium segment based minigenomes (M-minigenome) for AKV, OYAV, and Perdoes virus (in a previous publication we describe Perdoes virus as a reassortant containing OROV S and L segments [13]). The activity of these minigenome plasmids were then assessed using SBV, OROV or BUNV N and L proteins. Briefly, BSR-T7/5 cells were transfected as previously described and reporter activity, detected at 24 h p.t., OYAV and Perdoes virus M-minigenomes were functional with OROV, SBV and BUN N and L (Figure 5), however reporter activity from AKV M-minigenome could only be detected with SBV N and L (Figure 5B). The results also demonstrate that OROV and SBV N and L proteins can transcribe and replicate BUNV M-minigenome (Figure 5A,B), whilst BUNV N and L can transcribe and replicate OROV M-minigenome and to a less extent SBV M-minigenome (Figure 5C).

## 4. Discussion

Segmented viruses carry the potential for genome reassortment, an important characteristic as this allows these viruses to generate new strains and new virus species. This phenomenon, on occasions, can result in the acquisition of new or increased pathogenic characteristics and, hence, understanding molecular determinants driving reassortment can be beneficial for predicting the emergence of a new virus. Additionally, the information gathered by studying reassortment may be applied to vaccine development. There are currently no vaccines available for any member of the *Orthobunyavirus* genus and vaccines based on attenuated viruses carry the risk of reverting back to pathogenicity due to genetic exchange with naturally circulating wild-type strains. Hence, understanding factors that drive reassortment will allow such vaccines to be implemented effectively.

Using minigenome systems, the current study focuses on SBV and OROV, two important Simbu serogroup viruses of veterinary and public health importance. As expected, *Renilla* luciferase activity was detected in both the OROV and SBV medium segment based minigenome systems (i.e., using the N and L proteins corresponding to their medium segment analogue, M-minigenome), demonstrating the specificity of virus replication (Figure 1A). Unexpectedly, *Renilla* activity was also detected when SBV N and L proteins were assayed with OROV M-minigenome, but not the contrary. To confirm the minigenome results, a VLP assay was carried out. Bunyavirus assembly and budding occurs via interactions between the Gn-CT and the RNP [22,25], hence, this forms another important restricting factor for the emergence of new reassortant viruses. The VLP data demonstrated that OROV glycoproteins and SBV-based RNPs are capable of forming iVLPs, whilst SBV glycoproteins were unable to form transmissible/entry-competent VLPs with OROV-based RNPs (Figure 2A, OROVN + OROVL + SBVM). These results are important as: (i) they can be taken as confirmation that the minigenome assay results are valid, (ii) it supports the hypothesis that reassortant SBV-OROV viruses could potentially be generated, and (iii) it is likely that the potential reassortant virus would comprise of an OROV medium segment and SBV small and large segments. Our data also indicates a possible restrictive association between heterologous Gn and N proteins, suggesting that this interaction may be important for virus assembly. In phleboviruses (*Phenuiviridae* family), the Gn CT acts as a matrix protein, bridging the RNA and glycoproteins for RNP trafficking and virus assembly [25,29]. However, this specificity of the Gn CT and N protein/RNP interaction is not well documented in orthobunyaviruses (*Peribunyaviridae* family).

The difference between the medium segment UTR nucleotide positions eight and nine of the clade A Simbu viruses (OROV, OYAV, Perdoes) and the clade B Simbu viruses (SBV, AKV) is intriguing and it is tempting to speculate that these two residues may play a part in determining reassortment. The availability of complete orthobunyavirus sequences would be beneficial to allow a comprehensive analysis of this region and to determine if there is any relevance towards the virus–host pairing. For now at least, our results show that the N and L proteins of SBV exhibit a much broader UTR recognition range as it successfully transcribed and replicated AKV, BUN, OROV, OYAV and Perdoes virus M-minigenomes, unlike OROV or BUNV N and L (Figure 5; Table 1). This result would indicate that even though closely related viruses may share a degree of conservation in their N and L proteins, it is also the secondary structure of the medium segment UTR that drives their interaction. It has been demonstrated for other bunyaviruses that the interaction between the viral promoter and its polymerase is mediated by the nucleotide at position 8 of the 3′ UTR [3]. For La Crosse Virus (LACV), this interaction occurs at the “clamp” region of the L protein, which interacts with the adenine nucleotide at position 8. Interestingly, a majority of Simbu viruses including SBV and OROV possess a deletion of an asparagine residue in this region, which could possibly change the interaction between their L proteins and the promoter region of other bunyaviruses, reducing the specificity we observed in our results [3]. By mutating the eighth and nineth nucleotides of OROV M-minigenome (8A/T–9C/A) to mimic the residues of SBV M-UTR (8A/A–9A/A), we found that OROV N and L proteins lost their ability to recognize and transcribe their own OROV M-promoter, whilst the efficiency of SBV N-L decreased as well (Figure 3B). This is interesting because the downstream sequences in the OROV M-minigenome were unchanged, confirming previous results of the importance of this region in promoter activity [20,27,30]. Further, changing SBV M-minigenome (8A/A 9A/A) to mimic OROV medium segment UTR (8A/U 9C/A), with the rationale that if positions eight and nine of the M UTRs are determinants for promoter activity, then maybe changing the SBV M-minigenome at these positions could rescue minigenome activity with the OROV N and L proteins. However, this was not the case (Figure 3C; OROV N-L, 8U/A 9C/A) and, instead, minigenome activity decreased, even with SBV N and L proteins (Figure 3C, SBV N-L, 8U/A 9C/A). These results indicate that although the first 11 nucleotides are important, sequences beyond these residues are also essential for promoter activity. Previous work on BUNV has shown that mutant viruses lacking large portions of the UTR close to the coding-regions are highly attenuated due to diminished gene regulation [28,31,32].

Co-infection with bunyaviruses could theoretically result in eight potential genome combinations: AAA, ABA, BAA, AAB, BBA, ABB, BAB and BBB (where AAA and BBB are parental viruses and the combination arranged as SML segments) [7]. However, as the N (small segment product) and L (large segment product) need to be from the same virus for genome replication and transcription to occur, this substantially restricts viable reassortant combinations. The determining factor is then based on recognition of the medium segment UTR in virus B by the N and L proteins of virus A. Our data demonstrates that OROV N and L proteins are unable to use SBV medium segment promoter. Therefore, in the case of OROV and SBV, it is likely that the only progeny reassortant that could arise from these two viruses would contain SBV small and large segments with an OROV medium segment (as SBV N-L proteins can use both OROV and SBV medium segment UTR as a promoter). Based on these findings, out of the eight possible progeny viruses between OROV and SBV, there may in fact be only one viable new reassortant, i.e., a virus with the small and large segments from SBV and a medium segment from OROV. These results imply that the number of possible reassortants in nature may not be as vast as we think and we propose that reassortment may be driven by the medium segment.

Both OROV and SBV are transmitted by *Culicoides* midges, which have a wide geographic distribution [33]. The spread of SBV outside of Europe is a threat to the cattle farming industry and, in a setting such as Brazil, if geographic boundaries are broken, the possibility of OROV and SBV replicating in the same vector population could arise, with the risk of reassortment. Arboviral reassortants have been reported to arise in arthropod vectors, and it has been suggested that these insects could have played a major role in the evolution and emergence of RNA viruses [34,35]. Taken together, our data suggests that the emergence of a new reassortant virus containing the small and large segments of SBV and the medium segment of OROV could potentially be a cause for a SBV spillover into the human population.

## Figures and Tables

**Table 1:** A summary of the medium segment based minigenomes (M-minigenome) that are functional with either the N and L proteins of BUNV, OROV or SBV (✘ indicates that *Renilla* activity was not detected, ✔ indicates that *Renilla* activity was detected).

M-minigenome	N and L Proteins		
	BUNV	OROV	SBV
Akabane virus (AKV)	✘	✘	✔
Bunyamwera virus (BUNV)	✔	✔	✔
Oropouche virus (OROV)	✔	✔	✔
Oya virus (OYAV)	✔	✔	✔
Perdoes virus	✔	✔	✔
Schmallenberg virus (SBV)	inefficient	✘	✔
